# ML-Based Detection of DDoS Attacks Using Evolutionary Algorithms Optimization

**DOI:** 10.3390/s24051672

**Published:** 2024-03-05

**Authors:** Fauzia Talpur, Imtiaz Ali Korejo, Aftab Ahmed Chandio, Ali Ghulam, Mir. Sajjad Hussain Talpur

**Affiliations:** 1 Institute of Mathematics & Computer Science, University of Sindh, Jamshoro 70680, Sindh, Pakistan; fozia.g.talpur@gmail.com (F.T.); chandio.aftab@usindh.edu.pk (A.A.C.); 2 Information Technology Centre, Sindh Agriculture University, Tandojam 70060, Sindh, Pakistan; garahu@sau.edu.pk (A.G.); mirsajjadhussain@sau.edu.pk (M.S.H.T.)

**Keywords:** DDoS, XGB-GA, RF-GA, SVM-GA, TPOT, genetic programming, machine learning

## Abstract

The escalating reliance of modern society on information and communication technology has rendered it vulnerable to an array of cyber-attacks, with distributed denial-of-service (DDoS) attacks emerging as one of the most prevalent threats. This paper delves into the intricacies of DDoS attacks, which exploit compromised machines numbering in the thousands to disrupt data services and online commercial platforms, resulting in significant downtime and financial losses. Recognizing the gravity of this issue, various detection techniques have been explored, yet the quantity and prior detection of DDoS attacks has seen a decline in recent methods. This research introduces an innovative approach by integrating evolutionary optimization algorithms and machine learning techniques. Specifically, the study proposes XGB-GA Optimization, RF-GA Optimization, and SVM-GA Optimization methods, employing Evolutionary Algorithms (EAs) Optimization with Tree-based Pipelines Optimization Tool (TPOT)-Genetic Programming. Datasets pertaining to DDoS attacks were utilized to train machine learning models based on XGB, RF, and SVM algorithms, and 10-fold cross-validation was employed. The models were further optimized using EAs, achieving remarkable accuracy scores: 99.99% with the XGB-GA method, 99.50% with RF-GA, and 99.99% with SVM-GA. Furthermore, the study employed TPOT to identify the optimal algorithm for constructing a machine learning model, with the genetic algorithm pinpointing XGB-GA as the most effective choice. This research significantly advances the field of DDoS attack detection by presenting a robust and accurate methodology, thereby enhancing the cybersecurity landscape and fortifying digital infrastructures against these pervasive threats.

## 1. Introduction

The vast majority of the work in this area has focused on a Distributed Denial-of-Service (DDoS) assault. A DDoS is made possible when numerous computers are combined as an attack platform using client/server technologies, and then the attacks are launched at one or more targets to boost the attack’s potency. As an example of a DDoS attack, tens of thousands or even hundreds of thousands of compromised computers are utilized to target online businesses and information-providing services. This often results in substantial periods of inactivity and financial damages, as well as the denial of services to legitimate customers. The investigation of DDoS attacks is a prominent topic of research, and several methods for spotting DDoS attacks have been put forth in the literature, including evolutionary algorithms (EAs) and artificial intelligence. Hence, our suggested methodology can be employed to promptly identify Distributed Denial of Service (DDoS) attacks in real-time during their first stages. In addition, we employ a Genetic Algorithm (GA) to optimize the parameters utilized in the traffic matrix. The GA is a widely recognized heuristic method used to get the ideal value across a vast search field.

This strategy is centered on developing an intrusion detection system (IDS) that can discriminate between regular and attack traffic while also meeting the needs of the monitored environment. For our experimental analysis, the publicly accessible datasets KDD Cup 99 and CIC-IDS 2017 were employed. The utilization of several detection models enhanced the accuracy of detection, but at the cost of increased computational complexity. Several detection approaches have been presented in the field of statistical analysis. The last few years have seen an increased interest in GA, which was created by John Holland at the University of Michigan in 1975. The GA is based on genetics and natural selection concepts [1,2,3]. In computers, a genetic algorithm is a search strategy that is used to identify alternative solutions to optimize and search problems. A genetic algorithm uses evolutionary principles to find the best results that are close to the real ones. A genetic algorithm is an evolutionary algorithm that uses basic ideas to evaluate things in a way that is similar to how things work in nature. It has four main steps: determining the health of the population, reproducing, crossing over (recombination), changing a gene, and ending. In the crossover stage, two parents choose a good group and then mix (recombine) to make a new child.

## 2. Related Works

The GA automate the optimization of hyper-parameters, hence ensuring the efficient and effective detection of DDoS attacks. This study focuses on the difficulties related to the detection of App-DDoS assaults and proposes a highly efficient and flexible technique for identifying different forms of App-DDoS attacks [4]. DDoS attacks, in contrast to DoS attacks, are executed through a collaborative endeavor from multiple distributed sources, such as a botnet, to disrupt regular operations. The authors [5] (2020), and Kaur et al. [6] have published their respective works. HMGOGA addresses the limitations of traditional GOA, such as slow convergence speed and susceptibility to local optima. This work utilizes the proposed algorithm to identify DDoS assaults by means of simulating the combined nonlinear regression (NR)-sigmoid model [7]. The deployment findings demonstrate that the intrusion detection system, which utilizes a genetic algorithm, is capable of effectively detecting DDoS assaults on MANETs with high detection rates [8]. DDoS assaults, which produce large volumes of traffic, result in the depletion of network bandwidth and/or system resources. Hence, it is crucial to identify DDoS assaults at an early stage. This study presents an advanced method for detecting DDoS attacks [9].

We propose a method that combines information theory and genetic algorithms to identify anomalous network activities. By examining the reciprocal information between network properties and the types of network incursions, it is evident that a limited set of network features are strongly associated with network attacks [10]. The investigation of DDoS attacks is a substantial field of study. Numerous methods, such as EAs and artificial intelligence, have been suggested in the literature as a means of identifying DDoS attacks. Regrettably, the current widely recognized DDoS detection systems are declining in their ability to authenticate the intended purpose and previous identification of DDoS attacks [11]. Machine learning (ML) is becoming increasingly popular in the field of medicine, particularly in the areas of diagnosis and treatment management [12]. Numerous studies have been conducted to determine how ML can increase the timeliness and precision of diagnosis [13,14]. A crucial element of all healthcare systems around the world is accurate diagnosis. A mistaken diagnosis for a significant medical illness is given to about 5% of outpatients in the US [15]. Recently, reduced-space multistream classification based on Multi-objective Evolutionary Optimization has been proposed by researchers [16,17].

This paper employs neural networks for cloud resource consumption prediction with these factors in mind. Training the network weights is the main challenge in putting neural networks into practice [18]. The network’s weights training is a challenging optimization challenge. For these issues, swarm and EAs are frequently used. These techniques are favored over the usage of conventional mathematical approaches [19]. In [20], a meta-heuristic approach with immigrant techniques was proposed for nurse duty rosters in public hospitals in Sindh, Pakistan. The suggested model employs a hybrid GA and Particle Swarm Optimization (PSO) (GA-PSO) that capitalizes on the advantages of both techniques to accomplish this goal. Impressive results have been obtained when hybrid versions of these algorithms are utilized in a number of different disciplines. Antenna array pattern synthesis [21], mining association rules [22], forecasting power consumption [23], allocating resources in cloud computing [24], and process planning [25] are a few of the applications. The simulation used by the authors of [26] demonstrated that the hybrid version outperforms the drawbacks of the individual approaches [27]. In a cloud computing context, processing is done by cloud servers housed in data centers that offer infrastructure, software, and platforms as internet-based services rather than by local computers. In fact, cloud computing’s objective is to combine hardware and software as a service that is available to consumers via the Internet [28,29]. Popular cyberattacks include denial of service attacks, distributed denial of service attacks [26], remote to local attacks [30], probing attacks, user to root attacks, adversarial attacks, poisoning and evasion attacks, botnet attacks, phishing attacks [31], spamming attacks [32] and zero-day attacks [33]. There is a consensus that integrating GA with a machine learning technique called integrating evolutionary optimization algorithms can effectively mitigate denial of service threats. However, differentiating between a DDoS attack and regular traffic is challenging, since DDoS attacks frequently lack dangerous material in their packets. In addition, attackers manipulate their source addresses in order to obscure their whereabouts and enhance the complexity of DDoS attacks [11].

## 3. Materials and Methods

### 3.1. Datasets and Source

The proposed algorithm for datasets construction provides step-by-step directions on how to put together a dataset. The dataset has 22 features and may be accessed in the Mendeley data repository. The collection primarily consists of statistical features. Our dataset’s traffic flow encompasses three distinct protocols: TCP, UDP, and ICMP. The dataset is programmatically annotated by assigning labels to the traffic based on a variable that distinguishes between different traffic categories [34]. Datasets deal with removing valuable data from the data source. The dataset receives its annotations in a computerized way as a result of the application of coding logic. The programming is designed in such a way that the label column of the dataset is set to “0” when benign traffic is running, but is set to “1” when malicious traffic is running. There is a “1” in the traffic label. Following the annotation of the data, we then will classify the traffic using any machine learning algorithm. The dataset contains a comprehensive representation of traffic incidents, as seen in Table 1 [34].

### 3.2. Proposed Novel Hybrid Method for DDoS Attack Detection Using Tree-Based Pipelines Optimization Tool (TPOT) with Genetics Algorithm

This study, using qualitative techniques to analyze computer networks’ security, has benefited greatly from the use of DDoS network attacks. An interesting side finding was the ability to identify several attack vectors and instances of illegal software activity that firewalls may occasionally miss. Many DDoS attacks have been enhanced to classify network traffic as regular or abnormal using machine learning methods. In the proposed method, two phases of the new hybrid DDoS detection method—in the first phase for feature selection and a second phase for attacks detection—are described in this work.

**Figure 1:**
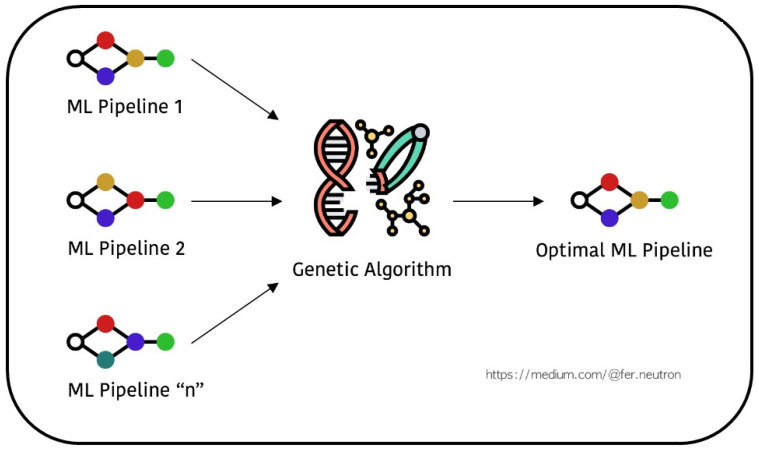
TPOT: Searching Pipelines Optimization with Genetic Algorithms.

We employed a supervised learning method for classification. These findings then suggested an integrated XGB algorithm with GA, RF algorithm integrated with GA, and SVM algorithm integrated with GA, used with Tree-based Pipelines Optimization Tool (TPOT) with GA be applied during the feature selection stage as shown in Figure 1. A further complication for the present hypothesis is that proposed and implemented initial scheme model frameworks used for building ML Pipeline 1, Pipeline 2, and the total number of Pipeline N as machine learning algorithms, were then integrated with genetic algorithms associated with the optimal ML Pipeline. An open-source AutoML tool called TPOT automates the pipeline optimization process for machine learning. TPOT assists in the automatic search for the optimal ML pipeline, which includes feature selection, model selection, hyperparameter tuning, and data preprocessing, using genetic programming [35].

At present, we have undoubtedly encountered the essential task of choosing the appropriate model, along with the appropriate parameters, and so on. Even so, this process can be challenging due to the extensive range of choices. Implementing GridSearch to identify the ideal settings for our proposed pipeline might be a highly time-consuming process. Tools like TPOT serve as assistants in the search for the most optimal pipeline as shown in Figure 1.

TPOT is an automated machine learning (AutoML) tool that is particularly developed to construct optimal pipelines using genetic programming in an efficient manner. TPOT is an open source library that utilizes scikit-learn components for tasks such as conversion of data, feature decomposition, feature selection, and model selection. 

**Figure 2:**
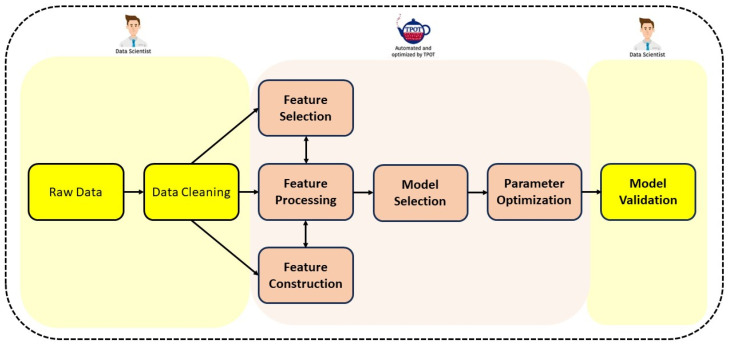
The components in a typical pipeline that are being examined by a Data Scientist are highlighted in yellow on both the right and left sides. The highlighted portion in the middle is an indication of the search for the optimal ML pipeline which is performed by TPOT.

While TPOT is categorized as an AutoML tool, it does not provide the complete “end-to-end” functionality of a machine learning pipeline. TPOT is primarily dedicated to the efficient automation of particular elements inside a machine learning pipeline. Figure 2 illustrates the phases that are automated by TPOT, as well as the phases that are particularly focused on by a Data Scientist or ML Engineer.

### 3.3. Three Machine Learning Algorithm Integration with Genetics Algorithms

All analyses using the XGB-GA optimization method, RF-GA optimization and SVM-GA optimization features such as multi-parent crossover and multi-parent mutation are combined in this method. An XGB-GA was utilized to detect network attacks during the attack detection phase. The classifier was trained using a hybrid XGB-GA optimization and genetic programing algorithm optimization (GAO) approach to enhance performance. The proposed hybrid approach combined the XGB-GA optimization method, RF-GA optimization, and SVM-GA optimization based on EAs optimization. 

Several findings of this study warrant further discussion, such as how well the suggested evolutionary model worked in terms of accuracy compared to seven other algorithms: ET-GA, KNN-GA, BernoulliNB-GA, GBoosting-GA, SGD-GA, MultinomialNB-GA, and LR-GA. The results show that the proposed XGB-GA, RF-GA, and SVM-GA methods can achieve a maximum detection accuracy of 99.00%. The dimension reduction occurred when we used the KDD datasets with 42 to 16 features, and the maximum training time was only 10 s. The KDD dataset was used as a standard to test the attack detection methods. 

Genetic programming is used by TPOT, a Python Automated Machine Learning tool, to optimize machine learning pipelines. TPOT finds the optimal pipeline for data by intelligently sifting through thousands of potential pipelines, automating the most laborious component of machine learning. Upon completion of its search (or when you give up waiting), TPOT gives you the Python code for the optimal pipeline it discovered so you may continue to modify the pipeline. Since TPOT is built on top of scikit-learn, users of scikit-learn should be able to recognize all of the code that it creates. This version of TPOT optimizes a machine learning pipeline using only XGBoost and the conventional set of pre-processing techniques.

### 3.4. The Framework of the Proposed DDoS Diagnosis Procedure

**Figure 3:**
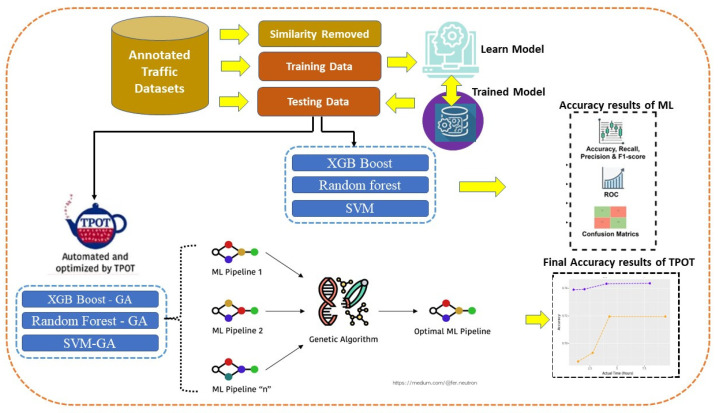
The framework of the proposed DDoS diagnosis procedure.

The general structure of the proposed diagnosing procedure is depicted for machine learning in Figure 3. Once we have the dataset, we may go on to the next framework in Figure 3 to see if any pre-processing is required to eliminate missing values or to replace them with suitable data for the genetic algorithm. Even though we could have eliminated the faulty rows of our datasets, we opted to fill in the missing values automatically by taking the average of the remaining ones. Following this step, GA is applied on the now-clean dataset in order to determine which subset of characteristics yields the highest correlation to the targets.

Our findings indicate that the random search frequently produced unnecessarily complicated pipelines for benchmark problems, even when a straightforward pipeline with a validated model was capable of accurately classifying the benchmark problem. Although random search may occasionally achieve similar accuracy to TPOT, conducting a guided search for pipelines that maximize accuracy while minimizing pipeline operations provides significant benefits in terms of search run-time, model complexity, and model interpretability.

Future studies will still be needed for the foreseeable future, although automated machine learning may speed up the process of finding effective models. Genetic programming is used by TPOT, a Python Automated Machine Learning tool, to optimize machine learning pipelines. It takes more than just fitting one model to the dataset to run TPOT. In a pipeline with multiple preprocessing steps (missing value imputation, scaling, PCA, feature selection, etc.), various machine learning algorithms (XGB, Random forests, SVMs, etc.) and their hyper-parameters are taken into account. Additionally, there are various ways to ensemble or stack the algorithms within the pipeline. Because of this, it typically executes slowly and is impractical for big datasets.

## 4. Evaluation Metrics

Many different Machine-Learning models have been trained and tested on our dataset. Several measures, such as Accuracy, Sensitivity, Specificity, Precision, and F1-score, were used to check how well each model worked. The true and false values of the classifier are displayed in a 2 × 2 matrix, which is the definition of the error matrix in a binary classification problem. Below is an explanation of the matrix’s four values, which can be somewhat perplexing at first glance [36].

- True Positive, TP: When both the model’s forecast and the actual values in the dataset are positive, we say that a value is a true positive, or TP, meaning the classifier accurately differentiates between good and bad traffic.
- True negative TN: When both the model’s forecast and the actual values in the dataset are negative, we say that the value is a true negative TN, i.e., it is the circumstance where the traffic is accurately categorized as malicious.
- False Positive, FP: False positive is the error category where the model prediction is positive but the actual value in the dataset is negative, i.e., it is the circumstance where the traffic is wrongly classed as innocuous.
- False Negative FN: A false negative is a form of error in which the actual values in the dataset contradict the prediction of the model, i.e., it is the circumstance where the traffic is wrongly categorized as harmful.
- As a performance metric, accuracy may be written as a fraction with the sum of correct answers (positive and negative) in the numerator and the sum of incorrect answers (positive and negative) in the denominator.

(1) $$\mathrm{Sensitivity}=\frac{\mathrm{TP}}{\mathrm{TP}+\mathrm{FN}}$$

(2) $$\mathrm{Specificity}=\frac{\mathrm{TN}}{\mathrm{TN}+\mathrm{FP}}$$

(3) $$Accuracy=\frac{TP+TN}{TP+FP+TN+FN}$$

(4) $$\mathrm{Precision}(p)=\frac{TP}{TP+FP}$$

(5) $$F1score=\frac{2*R*P}{R+P}$$

(6) $$\mathrm{MCC}=\frac{\mathrm{TP}*\mathrm{TN}-\mathrm{FP}*\mathrm{FN}}{\sqrt{\left(\mathrm{TP}+\mathrm{FP}\right)\left(\mathrm{TP}+\mathrm{FN}\right)\left(\mathrm{TN}+\mathrm{FP}\right)\left(\mathrm{TN}+\mathrm{FN}\right)}}$$

## 5. Results

### 5.1. Three Machine Learning Classification Results

We compared the SVM-RF hybrid model’s accuracy to that of many other well-defined machine learning models. Exploration of the data was performed to gain comprehension of the datasets, the distribution of normal and harmful traffic within the dataset, and the number of instances in each type of traffic. Table 1 below provides a concise summary of the analyzed dataset. A further complication for the present hypothesis is the determination of how much good and bad traffic makes up each type of traffic. To better understand the dataset some summary information is provided.

### 5.2. SVM

Once the dimensions have been minimized, SVC can be used to fit the data, as illustrated in Figure 3. This demonstrates that with repeated training on the dataset, the model becomes more accurate at classifying traffic. The model’s inability to appropriately capture the linear relationship between the features led to inaccurate predictions of the class labels, hence it does not provide adequate results. It also has extensive citations in the academic literature.

### 5.3. Random Forest (RF)

The classifier known as Random Forest (RF) makes use of many decision trees to reach a conclusion. It is possible for other decision trees to correct for an incorrect one. Each decision tree outputs a categorization result, with the highest scores being weighted toward the proposed ultimate score. This demonstrates that RF is the superior classifier.

### 5.4. XGBoost

Ensemble classifiers (ECs) are a type of classifier that combines the results of multiple classifiers into a single one. Classifiers like XGBoost, Random Forest, and SVM are used. The classifier has a 99.9% accuracy, which is significantly higher than the performance of separate classifiers.

Combining the strengths of SVC and RF, or “Support Vector and Random Forest”, creates a powerful new classification method. This classifier achieves the best results on our dataset when two machine learning methods are combined as shown in Table 2. This study used qualitative measures in order to determine the aforementioned efficiency metrics; the confusion matrix was frequently employed. The analysis was based on the dataset outlined in Table 2, with the results analysis consisting of calculated accuracy, precision, recall, and the F1-score to evaluate the system’s overall performance.

### 5.5. Receiver Operating Characteristic ROC (AUC) Training Performance

**Figure 4:**
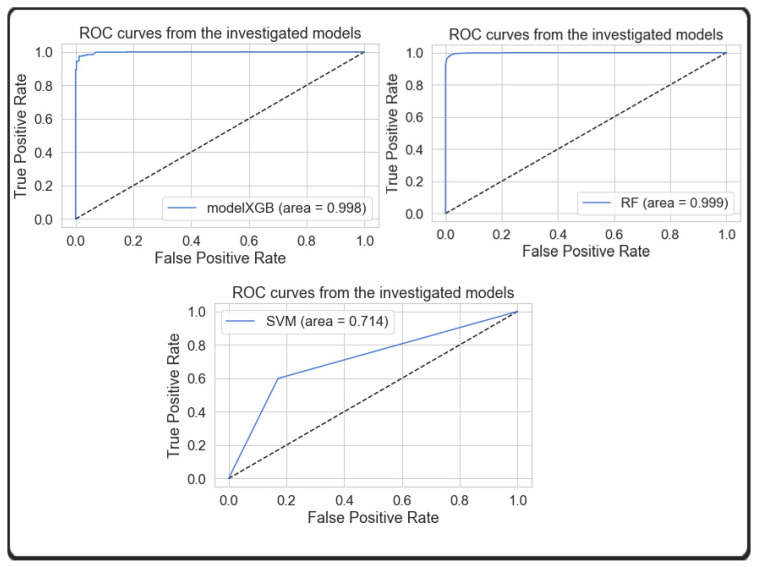
ROC(AUC) performance of a classification model.

This comparison analysis is further expanded to analyze the training performance of machine learning models through the use of k-fold cross validation and Area Under Curve (AUC) analysis of the Receiver Operating Characteristic (ROC) curves. The ROC curve is a tool that can be used to evaluate the performance of a classification model by taking into consideration the False Positive Rate (FPR) and the True Positive Rate (TPR). Figure 4 illustrates the ROC(AUC) graphs of XGBoost, Random Forest, and SVM, respectively.

### 5.6. Performing Accuracy Tests Using a Variety of Methodologies for Fivefold Cross Validation

As explained in the part titled “accuracy performance,” various different machine learning strategies were utilized in order to analyze the dataset. The dataset was subjected to five rounds of cross-validation, one of which was performed with each of these methods. It can be deduced from the results of the cross-validation that the XGBoost and RF models performed the best in terms of accuracy throughout training and testing. When it came to training, the Decision Tree performed in a manner that was approximatively comparable to XGBoost and RF. In the instance of the testing, the KNN performed poorly, having the highest number of deviations compared to the other methods.

## 6. Three Machine Learning Algorithm Optimizations with Genetic Algorithms Results

A more advanced technique to machine learning is used in AutoM, specifically, TPOT, to discover a passably efficient pipeline in our DDoS datasets. To enable TPOT to fully search the pipeline space in the DDoS datasets, it is frequently beneficial to run several instances of the program in parallel for a considerable amount of time (hours to days). AutoML algorithms involve more than just fitting a single model to the DDoS dataset; we have calculated a variety of machine learning algorithms (XGB-GA Optimization, RF-GA Optimization, and SVM-GA) in a pipeline that includes a number of preprocessing steps (like feature selection, scaling, PCA, missing value imputation), the hyperparameters for each model preprocessing step, and then we deployed a 5-fold validation method as a 5 Generation cross-validation using the pipeline arrangement options shown in Table 3.

We obtained the best pipeline test accuracy of 1.000 using the genetics algorithm. This demonstrates how a straightforward genetic algorithm implementation can enhance the performance measure in XGBoost. Two criteria were used to assess the performance of the pipelines: the trained pipeline’s classification accuracy and the training pipeline’s elapsed time as shown in Table 4.

It is generally agreed that computed MSE error rate in the missing numeric column values with the column’s most frequent value. Current research seems to indicate that the preferred solution is to divide the columns into categories and employ various imputation techniques according to whether the data were nominal, ordinal, or interval. Because not every column had a most frequent value, missing values in string columns were filled in with a “missing” label before they were ordinally encoded. Then, each feature would have an additional dimension created by TPOT’s single Gradient Boosting framework process, indicating that there was a missing value in the data for that feature. By default, TPOT Gradient Boosting framework employs mean squared error scoring.

The runs concluded rather fast, with distinct winning pipelines identified each time. The Root Mean Squared Error (RMSE) was obtained by taking the square root of the scores as shown in Table 5. Three methods were suggested for Hyper-Parameter Optimization (HPO) to improve the performance of the XGB classifier model, RF classifier model, and SVM classifier model and Genetic Programming (TPOT Classifier). The models, XG Boost, Random Forest, and SMV were evaluated against the results of previous research. All did a good job classifying the XGB-GA Optimization DDoS attack traffic, and we can identify benign traffic with a 100% accuracy rate. Compared to the other seven optimization methods using TPOT classifiers, XGB-GA Optimization performed well. XGB-GA Optimization accurately identified the DDoS attack traffic. The results of the best pipeline test accuracy score are also confirmed in Table 6.

### 6.1. RF-GA Optimization with Genetic Algorithms Results

In order to develop the model, we conducted all analyses using two algorithms, Random Forest-GA optimization and SVM-GA Optimization. The analysis was based on the contrast of each algorithm’s accuracy score. This helps us determine which is superior. This study used qualitative utilization of TPOT to create a machine learning model in the following section. TPOT first selects the best classification algorithm by combining all of the available algorithms. The utilization of the genetic algorithm optimization identified the algorithm that scored the highest on accuracy. The DDoS attack traffic dataset was used to train our model. Based on the input features, the model categorized the different types of attacks, as shown in Table 7.

Our model was constructed using the Random Forest Classifier, which is the second approach, integrated with the genetic algorithm RF-GA Optimization. Next, we computed the accuracy rating using contrast. After developing our model, we utilized TPOT to aggregate all of the methods to identify the optimal one. The accuracy scores of the two algorithms can then be compared. Accuracy ratings from RF-GA Optimization range from 0.9999 to 0.9960. Evidently, RF-GA Optimization is superior to SVM-GA optimization.

### 6.2. SVM-GA Optimization with Genetic Algorithms Results

SVM-GA optimization is the option researchers would select while creating the model. Nevertheless, since we have only compared two algorithms, this one could not be the best. Building models with various methods is a laborious procedure. TPOT is therefore the ideal option when working with many algorithms. TPOT finds the optimal classification method by combining all existing ones. As a result, it saves a much time by automating the genetic programming model building process and eliminating the need to manually compare every viable algorithm. SVM-GA optimization uses the same procedure for optimization. To identify the ideal pipeline, TPOT will iterate five times. This is beneficial since it automates the entire procedure, saving the users time. In this process of optimization, TPOT applies the theory of genetic programming.

It eventually determines the optimal algorithm as a result. We can also determine the precise parameters needed to accomplish this optimization with the aid of TPOT. We began by preparing our DDoS attack traffic dataset. Then, we employed two techniques to create a model utilizing this dataset. To determine which algorithm was superior, we compared two algorithms based on genetic programing. SVM-GA Optimization best pipeline test accuracy score was 0.9960, while the RF-GA Optimization best pipeline test accuracy score was 0.9999. Therefore, RF-GA Optimization proved to be the most effective algorithm through genetic programming, better than SVM-GA Optimization, as shown in Table 8.

### 6.3. Proposed Three TPOT-Classifiers with Other Seven GA Optimization Models Results

All four did a good job classifying the XGB-GA Optimization DDoS attack traffic, and we can both identify benign traffic with a 100% accuracy rate. Compared to the other seven optimization methods using TPOT classifiers, XGB-GA Optimization performed well. XGB-GA Optimization accurately identified the DDoS attack traffic. The results of the best pipeline test accuracy score can also be confirmed by the data in Table 6. ML algorithms were used to categorize the DDoS attack into several classes, and each category was then identified and verified according to different standards. A thorough examination of several GA Optimizations was done for the purpose of identifying DDoS multiclass cyberthreats, with XGB-GA Optimization method, RF-GA Optimization, and SVM-GA Optimization based on EAs Optimization using TPOT. The genetic programming reliability index of SVM-GA Optimization best pipeline test accuracy score of 0.9960, RF-GA Optimization best pipeline test accuracy score of 0.9950, and XGB-GA Optimization method best pipeline test accuracy score of 1.000, accomplished the goal as shown in Table 7. The comparison of results of many more types of DDoS attacks may be addressed for categorization and prediction in the future.

A recent line of research has focused on best pipeline test accuracy. We determined 1.000% best pipeline accuracy score based on the XGB-GA optimization method, 0.9950% best pipeline accuracy score based on the RF-GA optimization, and 0.9999% best pipeline accuracy score based on the SVM-GA optimization method. Finally, we used TPOT to find the best algorithm to use when building a machine learning model, which determined that the best algorithm was the XGB-GA algorithm.

## 7. Comparative Analysis with Existing Results

The present study employed a DDOS attack dataset to compare the paper’s proposed technique against previous research on DDOS attack detection in order to evaluate it (see Table 9). The best standard that is currently available was found to be 96% and the TPOT Best pipeline test accuracy was 1.000 It is evident from the data in Table 9 that the study’s suggested model has the highest accuracy. The EAs methodological paradigm that this study suggests is quite beneficial for identifying these attacks early on.

A comparison was made between the suggested approach in the paper and the previous research in the field of DDOS attack detection using emulated datasets. The highest benchmark result currently in use was confirmed to be 96%. As can be observed from Table 9, our suggested model improves this metric, achieving the highest accuracy of accuracy of 1.000. For the suggested model, the EAs method is important for attack detection. With a shorter training time, this model was found to be the highest performing model for our dataset.

## 8. Discussion

The aim was to evaluate TPOT’s capabilities and decide whether or not it should be incorporated into existing machine learning processes. The use of TPOT will enable it to choose the most applicable features from the original IDS dataset that can aid in distinguishing typical low-speed DDoS attacks and these features are then passed to classifiers such as the support vector machine, decision tree, nave Bayes, and multilayer perceptron to identify the type of attack. The simulation results show that EAs and ML classification methods will achieve good detection and accuracy with a low false-positive rate. To sum up, the use of TPOT to detect DDoS represents a promising advancement in automating the creation of machine learning workflows for cyber security through the use of EAs. The research design involved the field of automated machine learning is well-suited for EAs, and specific instruments like TPOT- DDoS accentuate the benefits of EAs by demonstrating how simple an EA solution can be. A dataset’s features can be used by machine learning algorithms to learn new things. For the purpose of this study, the dataset was used as a model for machine learning training. The traffic can be divided into classifications by the trained model: malicious and benign. To classify the traffic, the trained model can also be used in real-time.

This research utilizes the SDN-DDoS dataset [45] to train and test several deep learning techniques. Various hybrid deep learning methods, both supervised and unsupervised, are utilized for traffic classification. Among these, supervised algorithms yield notable outcomes. The main benefit of this research is the integration of innovative features for DDoS attack detection. New features were recorded in the CSV file to generate the dataset, and ML algorithms were trained using the resulting SDN dataset. The proposed Novel Hybrid Method for DDoS Attack Detection using TPOT with GA was employed for classification. The dataset utilized by the ML/DL algorithms comprises a compilation of publicly available datasets on DDoS assaults, alongside an experimental DDoS dataset created by us and openly accessible on the Mendeley Data repository. A Python application is responsible for categorizing traffic into specific types.

## 9. Conclusions

The present study employed a quantitative proposed XGB-GA Optimization method, RF-GA Optimization, and SVM-GA Optimization, based on EAs Optimization using TPOT- Genetic programming to find the highest score as the model accuracy. The networking architecture that software has defined is called software-defined networking, or SDN. The controller, which remotely guides the traffic between the hosts, centrally controls network traffic. Even with such adaptable network traffic management, the network is still vulnerable to a number of threats. In this research, the authors develop an SDN dataset and use machine learning methods to distinguish between traffic from DDoS attacks and benign traffic. To deal with uncertainty in cloud computing environments, it is important to be able to predict how cloud resources will be used. In cloud computing, users are given access to their applications from anywhere in the world via the Internet. Internet-based technology and online services are very important in the world of technology today. Services on the Internet are now a part of everyone’s daily life. This kind of service dependency has led to a new kind of change and has opened the door to attacks on network services. In DDoS attacks, multiple DoS attacks are launched against the victim (the destination server) at the same time by multiple infected systems acting as attack agents. This makes it so that a specific service is not available, by flooding the service provider’s resources with false requests, which is a huge risk for the network. Because of the difficulty in identifying DDoS attacks with the current countermeasures, many new techniques are needed to find and stop DDoS attacks more effectively. This approach is performed in two steps: firstly, initially features are selected through XGB-GA, RF-GA, and SVM-GA, and then the selected features are passed to the different classifiers XGB-GA, RF-GA, and SVM-GA, to classify the DDoS attack. Future research could focus on evaluating neural network predictors in other areas of cloud computing, such as predicting other resources like disc usage, cost-effectiveness, network, and lowering energy use for green computing. It would be helpful to support the proposed evolutionary neural network approach by working on other multivariate datasets of resource usage. The investigation of DDoS attacks is a prominent topic of research, and several methods for spotting DDoS attacks have been put forth in the literature, including EAs and artificial intelligence. Regrettably, current, well-known DDoS detection techniques are losing their ability to reliably identify DDoS attacks in advance and objectively. The present study employed a quantitative research method to obtain a 1.000% best pipeline accuracy score based on the XGB-GA optimization method, 0.9950% best pipeline accuracy score based on RF-GA optimization, and 0.9999% best pipeline accuracy score based on SVM-GA optimization method. Finally, this study used a combination of qualitative and quantitative analysis tools TPOT to find the best algorithm to use when building a machine learning model. Of the genetic algorithms, the best algorithm was the XGB-GA algorithm. We used ML algorithms and integrated with the TPOT-GA algorithm, for the first time used DDOS attack detection based on ML-GA to receive optimized results.

## Figures and Tables

**Table 1:** Traffic category of each traffic instance.

Traffic Class	Benign	Malicious
ICMP	24,957	16,364
TCP	18,897	10,539
UDP	22,772	10,816

**Table 2:** Performance Measures of different Algorithms.

Algorithms	Accuracy	Precision	Recall	F1-Score
SVM	72.00%	71.99%	83.99%	76.99%
Random forest	98.00%	98.85%	99.45%	98.01%
XGBoost	98.08%	99.78%	99.89%	98.99%

**Table 3:** Best pipeline: XGB-GA Optimization Accuracy Score (ML quality metrics).

Classifiers	Precision	Recall	F1-Score	Accuracy
Gradient Boost	1.00	1.00	1.00	99.99%
	1.00	1.00	1.00	

**Table 4:** XGB-GA Best metrics performance.

Classifiers	GA Generations	Best Internal CV Score	GA Optimization Best Accuracy Score
Gradient Boost	Generation 1	Current best internal CV score:	99.99%
	Generation 2	Current best internal CV score:	1.0
	Generation 3	Current best internal CV score:	1.0
	Generation 4	Current best internal CV score:	1.0
	Generation 5	Current best internal CV score:	1.0
		Best pipeline test accuracy:	1.000
		Accuracy:	99.99%

**Table 5:** Best pipeline: XGB-GA MSE Error rate.

Classifiers	MAE	MSE	R^2^
Gradient Boost	4.7917	4.7917	0.9997

**Table 6:** TPOT-Classifiers Optimization Comparison.

ML-GA Classifiers	5 Iterations/5-Fold CV	Best Pipeline Test Accuracy Score
Extra Trees Classifier	Internal cv score	0.8123
K-Neighbors Classifier	Internal cv score	0.8158
Bernoulli NB	Internal cv score	0.7322
GBoosting Classifier	Internal cv score	0.9910
SGD Classifier	Internal cv score	0.5283
Multinomial NB	Internal cv score	0.5307
Logistic Regression	Internal cv score	0.7151
SVM-GA Optimization	Internal cv score	0.9940
Best pipeline test accuracy	Internal cv score	0.9960
RF-GA Optimization	Internal cv score	0.9988
Best pipeline test accuracy	Internal cv score	0.9950
Proposed XGB-GA	Accuracy:	0.9999
	Best pipeline test accuracy:	1.000

**Table 7:** Best pipeline: RF-GA Optimization Accuracy Score.

Classifiers	GA Generations	Best internal CV Score	Best Pipeline Test Accuracy Score
RF-GA	Generation 1	Current best internal CV score:	0.9981
	Generation 2	Current best internal CV score:	0.9988
	Generation 3	Current best internal CV score:	0.9983
	Generation 4	Current best internal CV score:	0.9988
	Generation 5	Current best internal CV score:	0.9988
	Best pipeline test accuracy:		0.9988

**Table 8:** Best pipeline: SVM-GA Optimization Accuracy Score.

Classifiers	GA Generations	Best Internal CV Score	Best Pipeline Test Accuracy Score
SVM-GA	Generation 1	Current Pareto front scores:	0.9739
	Generation 2	Current Pareto front scores:	0.9835
SVM-GA	Generation 3	Current Pareto front scores:	0.9925
	Generation 4	Current Pareto front scores:	0.9925
	Generation 5	Current Pareto front scores:	0.9940
	Best pipeline test accuracy:		0.9960

**Table 9:** Comparison Results of traffic classification using various Simulated SDN Datasets.

S. No	Authors	Testing Accuracy
1	Meti et al., 2017 [37]	80%
2	Da Silva et al., 2016 [38]	88.7%
3	Perez-Díaz et al. [39]	95%
4	Ye et al., 2018 [40]	95.24%
5	Ko et al. [41]	96%
6	Han et al., 2018 [42]	96%
7	Myint Oo et al., 2019 [43]	97%
8	Auhoja, 2021 [44]	98.8%
9	Proposed XGB-GA Optimization	99.00%
10	Proposed TPOT Best pipeline test accuracy:	1.000%

## Data Availability

Data are contained within the article.
